# Design of an Embedded CMOS Temperature Sensor for Passive RFID Tag Chips

**DOI:** 10.3390/s150511442

**Published:** 2015-05-18

**Authors:** Fangming Deng, Yigang He, Bing Li, Lihua Zhang, Xiang Wu, Zhihui Fu, Lei Zuo

**Affiliations:** 1 School of Electrical and Electronic Engineering, East China Jiaotong University, Nanchang 330013, China; E-Mails: lhzhang@ecjtu.edu.cn (L.Z.); zgxiangyu@163.com (X.W.); fuzhihui@sohu.com (Z.F.); 2 School of Electrical Engineering and Automation, Hefei University of Technology, Hefei 230009, China; E-Mails: 18655136887@163.com (Y.H.); libinghnu@163.com (B.L.); 18655118171@126.com (L.Z.)

**Keywords:** temperature sensor, RFID tag, CMOS process, phase-locked loop

## Abstract

This paper presents an ultra-low embedded power temperature sensor for passive RFID tags. The temperature sensor converts the temperature variation to a PTAT current, which is then transformed into a temperature-controlled frequency. A phase locked loop (PLL)-based sensor interface is employed to directly convert this temperature-controlled frequency into a corresponding digital output without an external reference clock. The fabricated sensor occupies an area of 0.021 mm^2^ using the TSMC 0.18 1P6M mixed-signal CMOS process. Measurement results of the embedded sensor within the tag system shows a 92 nW power dissipation under 1.0 V supply voltage at room temperature, with a sensing resolution of 0.15 °C/LSB and a sensing accuracy of −0.7/0.6 °C from −30 °C to 70 °C after 1-point calibration at 30 °C.

## 1. Introduction

Radio Frequency Identification (RFID) and Wireless Sensor Networks (WSN) are the two key technologies of the Internet of Things. RFID is a technology to automatically identify objects or humans by utilizing radio waves and WSN is a technology to cooperate in network for monitoring environmental conditions by utilizing various sensor nodes. Recently research on the integration of RFID and WSN has been a hot topic [1]. RFID can extend the ability of a WSN by providing sensible properties to otherwise unsensible objects. WSN, on the other hand, being able to monitor physical events, can provide much more information for the measurement of temperature, humidity, pressure, *etc.*, than simple RFID. Moreover, in an RFID system, reader-tag communications are conducted in single hops without inter-communication among tags. RFID systems integrated with WSNs will enjoy the advantages of wireless multi-hop inter-communication over wider areas [2].

Integrating a RFID tag with sensor nodes is the main type of integration of RFID and WSNs. The passive RFID tag offers several advantages such as battery-less operation, high flexibility, low cost and fast deployment, which all result in its extensive applications in commercial use [3]. The passive RFID tag collects the wireless energy from the RFID reader as its power supply. Hence, the power dissipation performance, determining the maximum reading distance of the RFID reader, is crucial for the design of a passive RFID tag and its integrated sensor. Secondly, the occupied sensor area and its calibration effort should be minimized to maintain low cost. Thirdly, as the sensor is supplied by the on-chip power management unit, a good supply rejection is necessary to achieve accurate sensing. In recent years RFID tags integrated with temperature sensors have already introduced in several papers [4,5].

The traditional CMOS temperature sensors are based on the Bipolar Junction Transistor (BJT) [6,7,8,9]. These sensors convert temperature variation into a corresponding voltage, which is followed by an Analog to Digital Converter (ADC) to generate a digital output. However such sensors show power consumption in the mW range, which is unsuitable for passive RFID applications. Hence MOS-based temperature sensors targeted for wireless system have been introduced. For low power operation, time-to-digital [4,10,11,12] or frequency-to-digital conversion [5,13,14] is used instead of ADCs. The sensors consume less power than BJT-based sensors at the expense of resolution and accuracy. However, these sensors need an external clock as a reference, which deteriorates the power and accuracy performance of the sensors.

This paper presents a novel CMOS temperature sensor for RFID applications. The proposed temperature sensor employs a Phase-Locked Loop (PLL) architecture and can transfer the sensor values to a digital output in the frequency domain without a reference clock. The rest of the paper is organized as follows: Section 2 first introduces the PLL-based sensor architecture and then illustrates the circuit design of the temperature sensor in detail. The measurement results are presented and compared with previous designs in Section 3. Finally, some conclusions are drawn in Section 4.

## 2. PLL-Based Temperature Sensor Architecture

**Figure 1:**
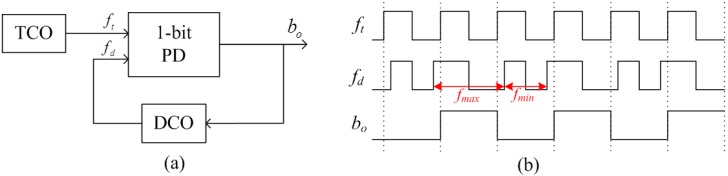
PLL-based temperature sensor; (**a**) architecture; (**b**) corresponding waveforms in locked state.

The proposed temperature sensor theory, developed from the PLL-based interface theory [15,16], is shown in Figure 1a. It consists of two main blocks: a frequency-modulating block, which converts the temperature information into the frequency domain, and a frequency-demodulating block, which converts the frequency into the digital domain, resulting in a complete temperature-to-digital flow. The frequency-modulating block consists of a Temperature-Controlled Oscillator (TCO) that directly converts the temperature information into a corresponding frequency *f_t_*. The frequency-demodulating block is a digital first-order PLL, consisting of a 1-bit Phase Detector (PD) and a Digitally-Controlled Oscillator (DCO). This PLL measures whether *f_t_* leads or lags *f_d_* (the output frequency of the DCO) and hence the DCO is only steered by a 1-bit signal *b_o_*. When the entire feedback loop is locked, *f_d_* shifts between a maximum and minimum value (*f_max_* and *f_min_*) which correspond to the maximal and minimal value of the temperature (seen in Figure 1b). The average value of *f_d_* will equal *f_t_*. Therefore, the over-sampled output *b_o_* represents the digital value of the temprature information.

The difference between these two frequencies defines the lock range of this first-order PLL. Rearrangement of the building blocks shows that this structure is similar to a single-bit first-order noise-shaped ∑△-modulator, but operating in the frequency domain instead of the voltage domain. The first-order PLL can be seen as a frequency-to-digital ∑△-modulator in which the frequency error is integrated and single-bit quantized, which means that the quantization noise is first-order noise-shaped to the output [17].

**Figure 2:**
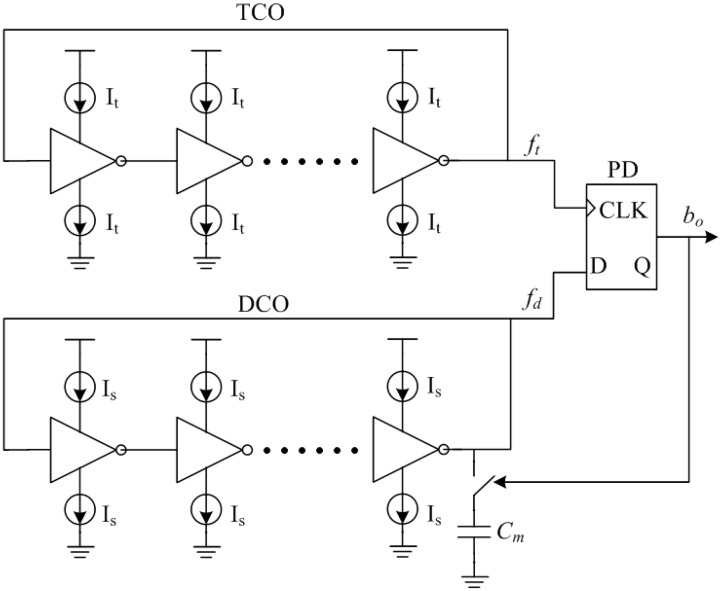
Proposed temperature sensor architecture.

The proposed temperature sensor architecture, based on the PLL theory, is shown in Figure 2. Both the TCO and the DCO are implemented as N-stage inverter-based ring oscillators. The oscillating frequencies of TCO and DCO depend on the time it takes a transition to propagate around the loop. For a standard ring oscillator and assuming an equal rise and fall time for the different stages respectively, the oscillating frequency *f_osc_* of the ring oscillator can be expressed as follows:

(1) $$f_{osc}=\frac{1}{t_{d}}=\frac{I_{l}}{V_{m}C_{l}}$$

where *t_d_* is the delay time of the loop, *I_l_* is the current flowing through the inverter, *C_l_* is the total output capacitor of the loop, and *V_m_* is the swing range of the output voltage which mostly equals to the supply voltage *V_DD_*. Because each inverter will add extra capacitance and delay time, the stage number N is determined by the frequency *f_osc_*.

In this design, the TCO and DCO inverters have identical schematics except for their current sources and adopt a current-starved architecture for lower power dissipation and higher temperature stability. According to Equation (2), if the current source of the TCO *I_t_* is a Proportional To Absolute Temperature (PTAT) current, then the oscillating frequency of the TCO *f_t_* is a PTAT frequency. The current source *I_s_* of the DCO is a constant current. Within the measured temperature range, the *f_t_* can be considered as consisting of two parts: the quiescent frequency *f_to_* and the variable frequency *f_tm_*. The DCO is steered by the PD output signal *b_o_*, which is a representation of the phase difference between the TCO and the DCO. The variable capacitive load on a single stage of the DCO consists of a switch and a capacitor *C_m_*. The capacitor *C_m_* is swapped in or out of the DCO depending on the output *b_o_* from the PD. When *C_m_* is disconnected from the DCO, the oscillating frequency of the DCO *f_d_* is designed to be equal to *f_to_*. When *C_m_* is connected to the DCO, *f_d_* is designed to be slightly larger than *f_t_*. The PD is simply composed of a 1-bit d-flip-flop. When the entire feedback loop is locked, the average value of *f_d_* will correspond to the *f_t_*. Therefore, the *b_o_* represents the digital value of the corresponding temperature.

This proposed temperature sensor has a simple architecture and can adopt fully-digital blocks, resulting in low power dissipation and low chip area. Furthermore, this architecture can convert the temperature information into digital signals without an extra reference clock, resulting in improved accuracy performance. The conversion is based on locking two oscillators, which means that the relative frequency is of importance and not the absolute frequency. Hence any influence such as supply voltage and temperature variations will be canceled out if the two oscillators are completely matched. The matching between the TCO and DCO plays an important role in this design. Mismatch between the TCO and DCO can be expressed as an offset between the coefficients of the polynomials of the characteristics of the oscillators, resulting in a deterioration of the Effective Number Of Bits (ENOB). Considering the issues of system linearity and process variation, *C_m_* is normally designed as a programmable capacitor.

## 3. Proposed Temperature Sensor Circuit Design

**Figure 3:**
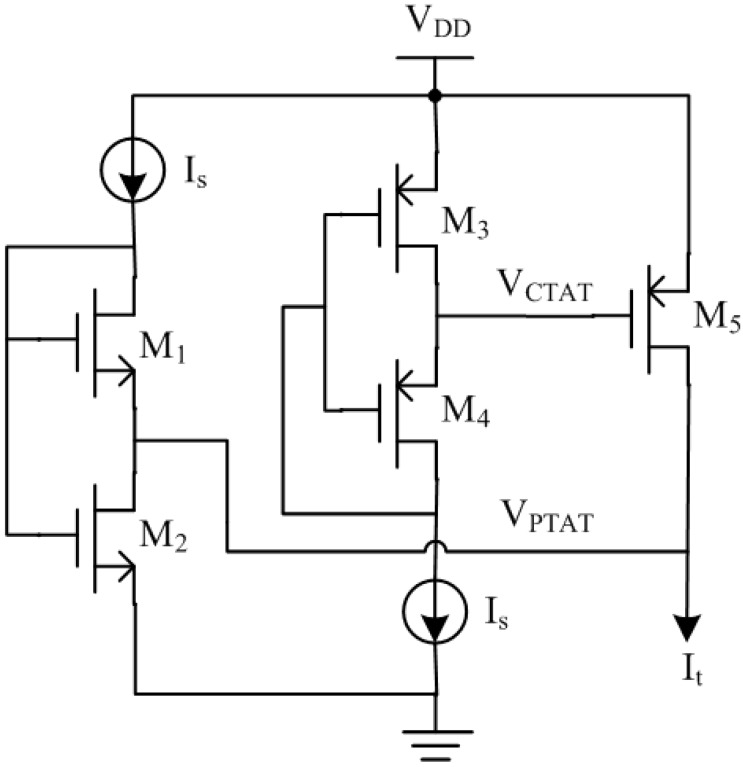
Proposed PTAT current source.

The key design of the proposed temperature sensor in Figure 3 is to generate a PTAT current source *I_t_*. Inspired by Amaravati [18], we design a Process and Voltage (PV)-compensated PTAT current source as shown in Figure 3. The transistors M_1_ and M_2_ are used to produce a PTAT voltage *V_PTAT_*, which is then applied on a PV-compensated resistor to produce the PV-compensated current *I_t_*. The transistors M_3_–M_5_ are used to produce this PV-compensated active resistor.

The NMOS transistors M_1_ and M_2_, biased in the subthreshold region, are short channel and long channel transistors respectively. The PTAT voltage *V_PTAT_* can be obtained as follows [19]:

(2) $$V_{PTAT}=V_{GS1}-V_{GS2}=\eta V_{T}\ln K$$

where *V_GS_*_1_ and *V_GS_*_2_ are the gate-source voltages of the M_1_ and M_2_ respectively, η is the subthreshold slope factor with an approximate value of 1.45, *V_T_* is the thermal voltage and *K* is the width ratio of this two transistors. The PMOS transistor M_5_ works in the triode region as an active resistor, which resistance can be expressed by:

(3) $$R=\frac{1}{\mu_{p}C_{ox}\left(W/L\right)\left(V_{SG}-\left|V_{th}\right|\right)}$$

where µ*_p_* is the hole mobility, *C_ox_* is the oxide capacitance of the transistor, *W*/*L* is the ratio of the width respect to the length of the transistor, *V_SG_* is the source-gate voltage of the transistor and *V_th_* is the threshold voltage. The effect of temperature variation of on µ*_p_* the required gate voltage is larger than that of *V_th_*. Hence we need a gate voltage with Complementary To Absolute Temperature (CTAT) behavior to compensate the effect of temperature variation. The PMOS transistors M_3_ and M_4_ are biased in the subthreshold region and the voltage *V_CTAT_* can be expressed as follows:

(4) $$V_{CTAT}=V_{DD}-\left(V_{SG4}-V_{SG3}\right)=V_{DD}-\eta V_{T}\ln K$$

**Figure 4:**
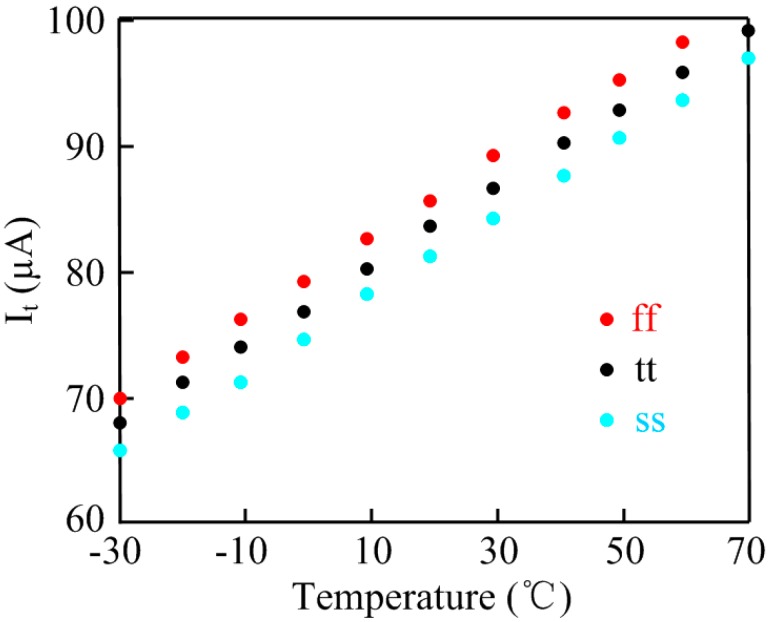
Simulation results of current *vs.* temperature on different process corners.

Equation (5) shows that the produced voltage is a CTAT voltage which also tracks supply voltage variations. Hence the output current *I_t_* is the PV-compensated PTAT current source. The simulation results of the proposed PTAT current source on different process corners are shown in Figure 4. The *I_t_* achieves good linearity with temperature and the simulated worst case variation across corners is ±4.5%.

**Figure 5:**
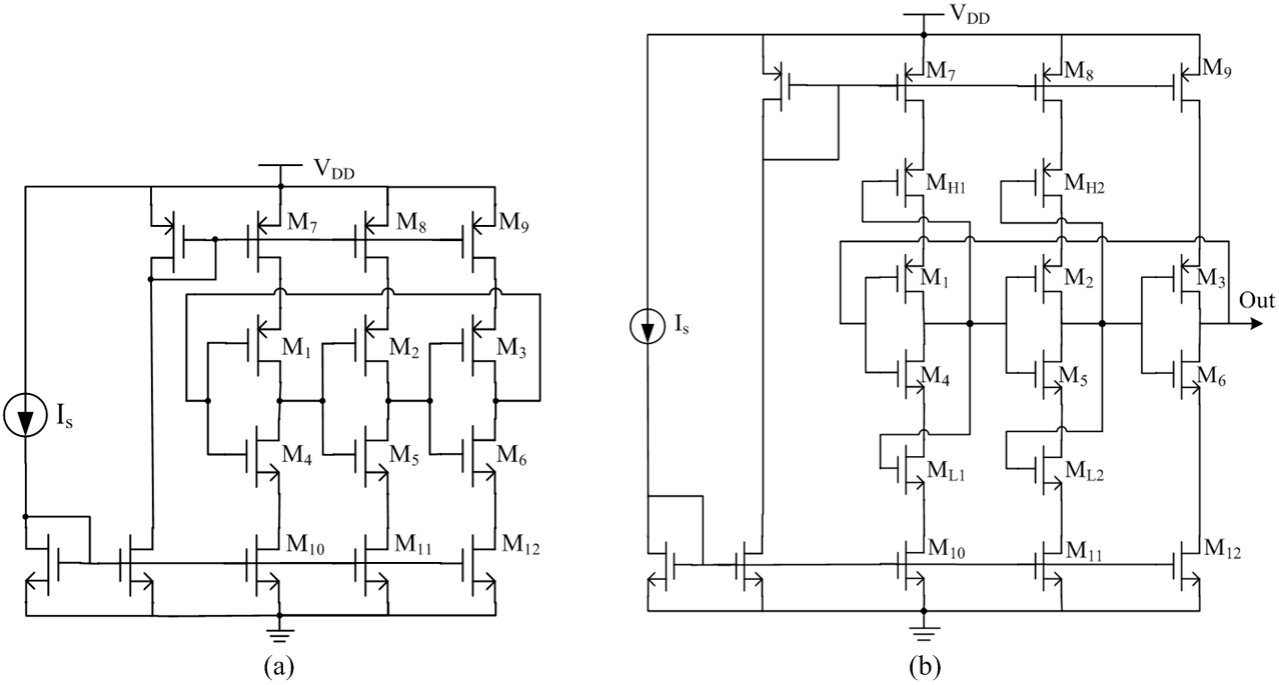
Current-starved ring oscillator; (**a**) conventional schematic; (**b**) proposed schematic.

In our design, the stage number of the TCO and DCO is selected as 3. In a conventional ring oscillator (shown in Figure 5a), the transistors M_1_–M_6_ form the three-stage inverters and the transistors M_7_–M_12_ form the current mirrors. The output nodes of all inverter cells experience a rail-to-rail swing from ground to the supply voltage *V_DD_*. However in the proposed method (shown in Figure 5b) [20], four extra transistors (M_H1_, M_H2_, M_L1_, M_L2_) are employed to constrain the voltage swing of internal nodes for reducing the overall power consumption, while the output node is left untouched to vary between the supply voltage and ground [21].

The four extra transistors (M_H1_, M_H2_, M_L1_, M_L2_) operate as follows: when the input switches from high-to-low, the inverter’s output starts to rise from low-to-high. As the output voltage rises, the gate voltages of M_H1_ and M_L2_ increase, which results in lowering the gate-source voltage of M_H1_ and M_L2_, *V_GSH_*. When *V_GSH_* falls below the threshold voltage and then M_H1_ and M_L2_ enter the subthreshold region where the current decreases exponentially. Therefore we can conclude the voltage headroom of the internal inverters is reduced to

(5) $$V_{DD}-\left(V_{GSH}+V_{GSL}\right)-\left(V_{DSH}+V_{DSL}\right)$$

where *V_DD_* is the supply voltage, *V_GSL_* is the gate-source voltage of M_L1_ and M_L2_, *V_DSH_* is the drain–source voltage of M_7_ and M_8_ and *V_DSL_* is the drain–source voltage of M_10_ and M_11_.

**Figure 6:**
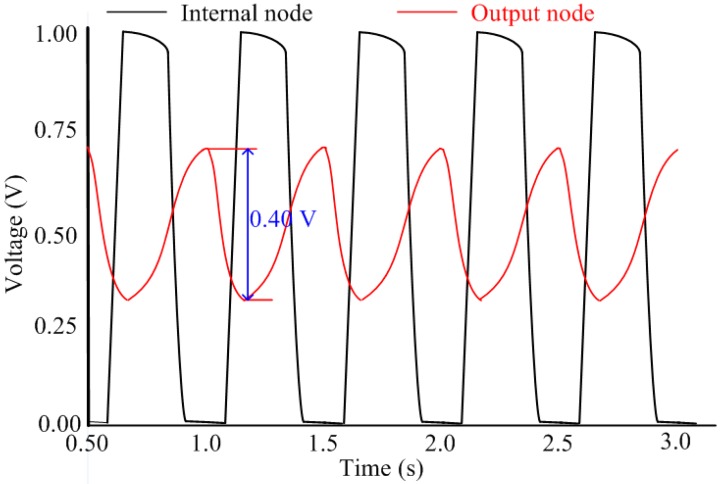
Output waveforms of the proposed ring oscillator at different nodes.

The simulation results, shown in Figure 6, indicate that the output node swings between the supply voltage 1.0 V and the ground, while the internal nodes vary from 0.3 V to 0.7 V. The reduction of the voltage swing to nearly 50% of its nominal value lowers the dynamic power consumption of the internal nodes by 75% and the overall power consumption by much more than 25%.

## 4. Measurement Results

**Figure 7:**
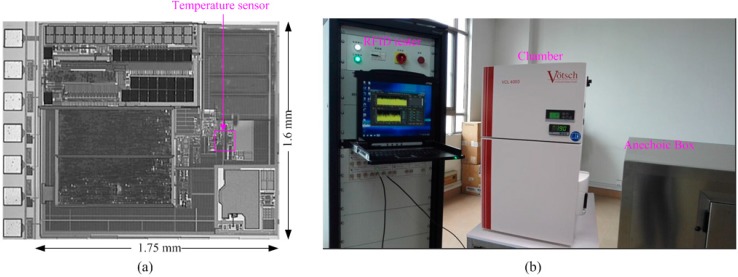
(**a**) Chip micrograph of the complete RFID tag; (**b**) Measurement equipments.

The RFID tag with the proposed temperature sensor was implemented in the Taiwan Semiconductor Manufacturing Company (TSMC) 0.18 μm 1P6M mixed-signal CMOS process. As shown in Figure 7a, the RFID tag chip covers an area of 1.75 × 1.6 mm^2^ without pad and the temperature sensor occupies an active area of 0.021 mm^2^. Extensive matching is exercised during the layout design stage in order to minimize the effects due to process variation and mismatch. In order to characterize the performance of the temperature sensor, the measurement is performed inside a VCL 4000 temperature chamber from Votsch Industry Electronics (Taichang, China), together with a calibrated thermometer as a reference. The output of the sensor tag is compared with the reference thermometer to analyze the linearity. The wireless test environment consists of a special RFID test instrument, a VISN-R1200 from VI Service Network (Shanghai, China) and an anechoic box (seen in Figure 7b). The VISN-R1200 can work as an adjustable RFID reader and display the testing signals simultaneously. The RFID tag was tested in the anechoic box for the electromagnetic shielding.

**Figure 8:**
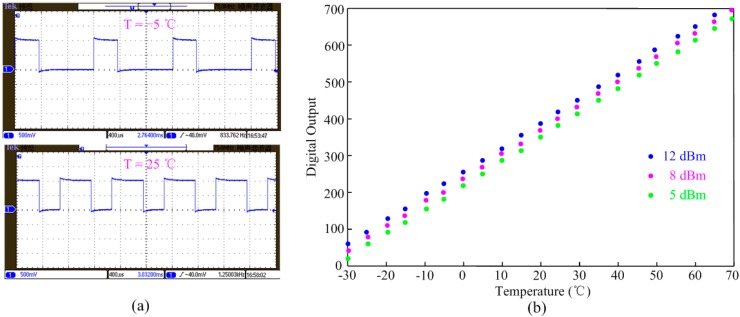
Performances of the test temperature sensor; (**a**) corresponding outputs of the sensor interface at −5 °C and 25 °C; (**b**) digital output of baseband *vs.* temperature.

Figure 8a shows the measured different outputs of the temperature sensor at −5 °C and 25 °C. It is easily concluded that the proposed temperature sensor shows higher oscillation frequency and average output duty cycle at 25 °C. 

The output of the temperature sensor is further processed by the digital baseband block in the RFID tag. The relation between the digital output of baseband and the temperature variation is shown in Figure 8b. The sensor tag was connected to the signal generator output and was tested under three different input power levels: 12, 8 and 5 dBm, respectively. The test sensor achieves about 0.15 °C /LSB resolution with a 1 kHz conversion frequency and high linearity performance within the measured range from −30 °C to 70 °C. The error of the linearity under different input power is small, which proves that the proposed temperature sensor has good immunity to supply voltage variation.

**Figure 9:**
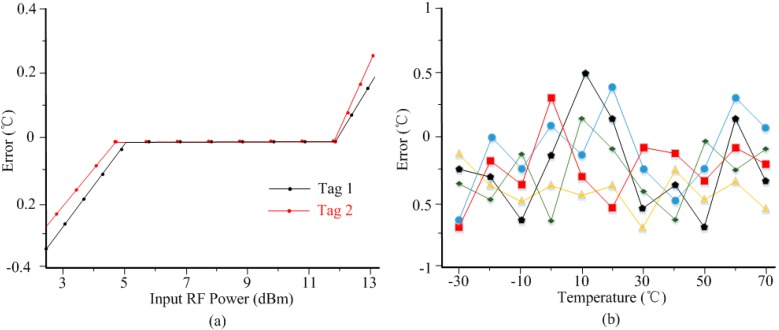
Measured sensing errors performance; (**a**) for different input RF power; (**b**) error curves for 5 test chips after 1-point calibration.

Because the sensor tag needs RF power to activate the internal building blocks, input power variation might affect the temperature sensor performance. Figure 9a shows the measured errors of two sensor tags under different input RF power levels. When the input power is small (≤5 dBm), the power received by the tags is not adequate to activate the tags, leading to the obvious sensing error that is observed. By increasing the input power, the sensor output becomes stable from 5 dBm to 12 dBm. If input power is too high (≥12 dBm), the internal power-limiter will convert the excess power into heat resulting in obvious errors. Figure 9b gives the measured temperature sensor inaccuracy over five different test chips. After 1-point calibration at 30 °C by adjusting the capacitor *C_m_*, the measured error is −0.7/0.6 °C from −30 °C to 70 °C.

Table 1 compares the measured performances of the proposed design wither other state-of-the-art designs, which have different performances and different target applications. The designs in [7,9] achieve high resolution and error performance, but consume too much power, thus resulting unsuitable for passive RFID applications. The designs in [11,12,13] achieve ultra-low power dissipation at the expense of resolution and error performance. Though the design in [14] obtains excellent power and resolution performances, the operating temperature range is small. The measurement results show the proposed temperature sensor covers 0.021 mm^2^ and draws 92 nA current from 1 V supply voltage. Furthermore, due to the immunity to supply voltage variation, this work requires only 1-point calibration, which ensures low-cost applications.

**Table 1:** Performances comparison of temperature sensor.

Reported Sensor	Process (µm)	V-Supply (V)	Power (nW)	Area (mm^2^)	Resolution (°C/LSB)	Range (°C)	Error (°C)
This work	0.18	1.0	92	0.021	0.15	−30~70	−0.7/0.6
[7]	0.065	1.2	10000	0.100	0.03	−70~125	−0.2/0.2
[9]	0.16	1.5	5100	0.080	0.02	−55~125	−0.15/0.15
[11]	0.18	0.5,1	119	0.042	0.2	−10~30	−0.8/1
[12]	0.18	1.0	405	0.032	0.3	0~100	−0.8/1
[13]	0.18	1.8	900	0.200	0.5	27~47	−1~1
[14]	0.35	1.4,2.1	110	0.084	0.04	35~45	−0.1~0.1

## 5. Conclusions

An embedded temperature sensor in a passive RFID tag is designed, measured and demonstrated in this paper. The temperature sensing part of the sensor converts the temperature variation into a PTAT current, which is then transformed into a temperature-controlled frequency. The sensor interface, based on a PLL architecture, can transform the temperature-controlled frequency into the corresponding digital output directly without an external reference clock. The fabricated sensor occupies an area of 0.021 mm^2^ using the TSMC 0.18 1P6M mixed-signal CMOS process. The measurement results show that the proposed sensor achieves 92 nW power dissipation with an error −0.5/0.6 °C from −30 °C to 70 °C after 1-point calibration. We will work on improving the resolution of this design in future. 
